# Supplementary material for: Impact of Rice Bran Oil Emulsified Formulation on Digestion and Glycemic Response to Japonica Rice: An In Vitro Test and a Clinical Trial in Adult Men
Source: Foods. 2024 Aug 21;13(16):2628. doi: 10.3390/foods13162628 (PMC11354130; doi:10.3390/foods13162628)
Supplement: Supplementary file 1 [file foods-13-02628-s001.zip › foods-3123510-supplementary.pdf]

foods MDPI

Patents

Supplementary Materials:

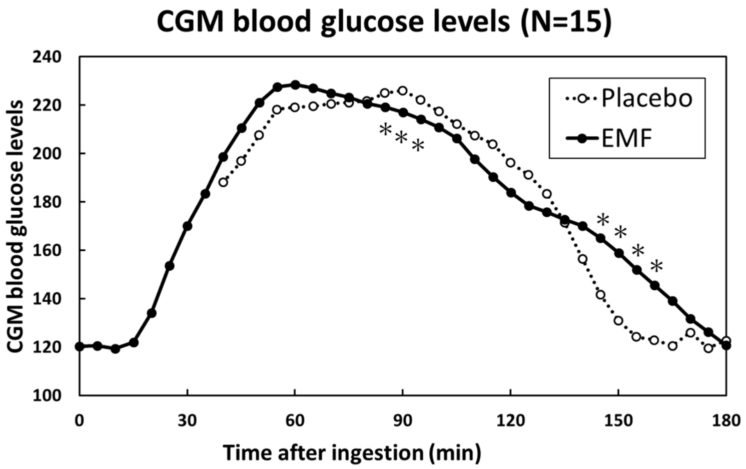

Supplemental Figure S1 Time course of CGM blood glucose levels at every five minutes are shown. The sample size at 170 min is due to the lack of data. * significant difference between placebo and EMF test foods
